# French national diagnosis and care protocol (PNDS, protocole national de diagnostic et de soins): cystic lymphatic malformations

**DOI:** 10.1186/s13023-022-02608-y

**Published:** 2023-01-13

**Authors:** Nicolas Leboulanger, Annouk Bisdorff, Olivia Boccara, Anne Dompmartin, Laurent Guibaud, Christine Labreze, Jacques Lagier, Bénédicte Lebrun-Vignes, Denis Herbreteau, Aline Joly, Julie Malloizel-Delaunay, Arnaud Martel, Stéphane Munck, Frédérique Saint-Aubin, Annabel Maruani

**Affiliations:** 1 Otolaryngology - Head and Neck Surgery Department. National Reference Center for Rare Otolaryngological Malformations (MALO), Necker Enfants Malades Hospital, 149 Rue de Sèvres, 75015 Paris, France; 2 INSERM U955, Paris Cité University. ERN Cranio, Paris, France; 3 Department of Interventional Radiology, Lariboisière Hospital, Paris, France; 4 Department of Dermatology and Reference Center for Rare Diseases and Vascular Malformations (MAGEC), Necker Enfants Malades Hospital, Paris, France; 5 Department of Dermatology, CHU Côte de Nacre, Caen, France; 6 Department of Radiology, Hôpital Mère-Enfant, CHU de Lyon, Lyon, France; 7 Department of Dermatology, Pellegrin Hospital, CHU de Bordeaux, Bordeaux, France; 8 Department of Ophthalmology, CHU de Nice, Nice, France; 9 Pharmacovigilance Unit, AP-HP, Department of Pharmacology, Pitié-Salpêtrière Hospital, Paris, France; 10 Department of Neuroradiology and Interventional Radiology - Reference Center for Rare Diseases and Vascular Malformations (MAGEC), CHRU de Tours, Tours, France; 11 Department of Maxillofacial Surgery - Reference Center for Rare Diseases and Vascular Malformations (MAGEC), CHRU de Tours, 37044 Tours, Cedex 9 France; 12 Department of Vascular Medicine, Rangueil Hospital, CHU de Toulouse, Toulouse, France; 13 Département d’enseignement et de Recherche en Médecine Générale, Retines, Healthy, Université Côte d’Azur, 06000 Nice, France; 14 Association AMLA (Agir Pour Les Malformations Lymphatiques), Presles-en-Brie, France; 15 Department of Dermatology and Reference Center for Rare Diseases and Vascular Malformations (MAGEC), CHRU de Tours, Tours, France; 16 INSERM 1246 ‑ SPHERE, Universities of Tours and Nantes, 37000 Tours, France

**Keywords:** Cystic lymphatic malformations, Vascular anomalies, Complications, Treatment

## Abstract

Cystic lymphatic malformations (LMs) are rare chronic conditions which management differs according to the type (macrocystic LMs, microcystic LMs or both). Studies are lacking due to rarity of the pathology. We aimed to establish a French National Diagnosis and Care Protocol (PNDS: Protocole National de Diagnostic et de Soins), to provide health professionals with free open access synthesis on optimal management and care of patients with LMs (https://www.has-sante.fr/upload/docs/application/pdf/2021-03/malformations_lymphatiques_kystiques_-_pnds.pdf). The process included a critical review of the literature and multidisciplinary expert consensus. LMs are congenital but are not always discovered at birth. Nearly 75% of them are located in the head and neck because of the highly dense lymphatic system in this region. Physical examination (showing painless masses with normal skin color and depressible consistency, or cutaneous/mucosal lymphangiectasia) and color Doppler ultrasonography, usually allow for diagnosis. MRI (involving T2 sequences with fat saturation in at least two spatial planes) is the tool of choice for evaluating anatomical extension, characterizing lesions (microcystic and macrocystic), and before considering therapeutic management. A biopsy, coupled to a blood sample, can also be used for molecular biology analyses, to search for activating mutations of the *PIK3CA* gene, particularly with LM integrating in a syndromic form (CLOVES or Klippel-Trenaunay syndrome) but also in certain isolated (or common) LMs. The spontaneous evolution of LMs, in particular microcystic forms, is often toward progressive aggravation, with an increase in the number of vesicles, thickening, increased oozing and bleeding, while pure macrocystic LMs may regress due to “natural sclerosis”, i.e. fibrosis secondary to an inflammatory reorganization after common infantile infections. In case of voluminous LMs or syndromic forms, functional and psychological repercussions can be major, deteriorating the patient’s quality of life. LMs must be treated by physicians integrated in multidisciplinary teams, and be personalized. Management is a life-long process that involves one or several of these therapies: conservative management, physical therapy (compression), sclerotherapy, surgery, drugs such as mTOR inhibitors (sirolimus), that has shown efficacy in decreasing the volume of LMs, and, more recently, PI3K-inhibitors in syndromic forms. Psychological and social support is necessary, taking into account the patient and his family.

## Background

Cystic lymphatic malformations (LMs) are rare chronic conditions with extremely heterogenous clinical features. Their management is also very variable depending on the characteristics, location, and natural history of the lesions. Treatment often includes a combination of conservative management, sclerotherapy, surgery, specific drugs, and should always be decided after multidisciplinary discussion. The purpose of this article based on a multidisciplinary French expert consensus is to present a state-of-art overview of currently available options for the management of LM.

## Objectives and development of the French national diagnosis and care protocol (Protocole National de Diagnostic et de Soin, PNDS)

In France, the management of rare diseases is ensured by several reference networks throughout the country. These networks provide care and orientation for patients as well as coordination of research and development of educational programs for health professionals. Different reference networks can cooperate and pool their expertise, as illustrated by the present recommendations on LM. The websites of the different networks involved in the writing of this document can be consulted here: https://fimarad.org/ for FIMARAD (rare dermatologic diseases and coordinator of the present study); https://favamulti.fr/ for FAVAMULTI (rare vascular diseases with multisystemic components); https://maladiesrares-necker.aphp.fr/magec/ for MAGEC (rare skin and mucosa diseases with a genetic origin) and https://maladiesrares-necker.aphp.fr/malo/ for MALO (rare head and neck malformations). The authors, members of the different networks involved in the management of lymphatic malformations, participated in the writing of this document. These recommendations have been established after an extensive review of the literature.

The objective of the present expert recommendations is to explain to the professionals concerned, and especially to the general practitioners, the current optimal diagnostic and therapeutic management and the care pathway for a patient affected with simple (cystic) LM. Their aim is to optimize and harmonize the management and follow-up of the rare disease throughout the country. They also make possible to identify pharmaceutical specialties used in an indication not provided for in the marketing authorization as well as specialties, products or services necessary for patient care but not usually covered or reimbursed by social security and/or private insurances. This PNDS is designed to be used as a reference by the physician at each step of the patient’s management. It aims to provide a clear and understandable state-of-the-art synthesis of the current knowledges and existing protocols on lymphatic malformations. Taking into consideration most of the possible clinical presentations, this document does not aim to define a single possible decision tree, but to present all current management possibilities: it cannot consider all the specific cases and therapeutic particularities. It describes the possible options in the reference management of a patient with a lymphatic malformation in 2022.

**Fig. 1:**
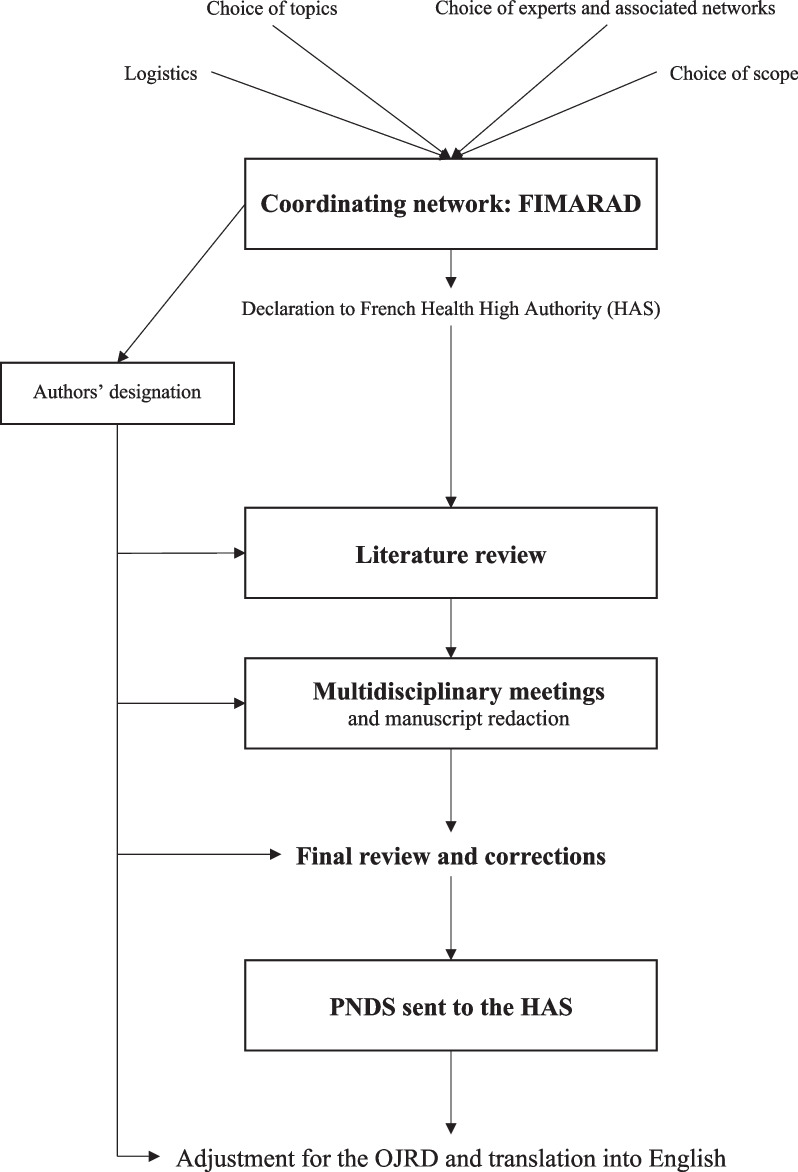
Development of a PNDS as recommended by the French National High Health Authority (HAS)

The methodology followed can be found here https://www.has-sante.fr/jcms/c_1340879/fr/protocoles-nationaux-de-diagnostic-et-de-soins-pnds (available in French) and is briefly summarized below and in the Fig. 1.

The coordinating network chose the group of experts, all belonging to the reference networks involved in the management of LMs and from different medical specialties. These experts performed an extensive literature review in their fields and wrote the synthesis document. The pooling, analysis and correction of the data was done during several meetings with the experts and the coordinating team. The final document has been corrected, reviewed and approved by all the co-authors and ultimately sent to the health authorities for online publication.

### Definitions

Cystic lymphatic malformations (LMs) are rare benign congenital slow-flow anomalies that feature abnormal cystic dilatations of lymphatic vessels [1–3]. The term “lymphangioma” has currently been dropped. The lymphatic system is a network of vessels that circulate parallel to the venous system, with which it shares a common mesodermal origin [4]. It drains lymph fluid, which carries high-molecular-weight molecules across the body.

LMs, present at birth as subcutaneous or deep soft masses that develop in proportion to the child's growth [2], are cystic, separated by septa. They are congenital but are not always discovered at birth: 90% of lesions show accelerated growth before age 5 years and can be revealed by inflammatory or infectious outbreaks [1]. LMs can be localized to the skin, the mucous membranes or underlying tissues (superficial LMs) or affect underlying organs (deep LMs). Nearly 75% of LMs are located in the head and neck because of the highly dense lymphatic system in this region [3].

Depending on their size estimated clinically or on imaging, LMs are said to be macrocystic (diameter > 1 cm), microcystic (< 1 cm and often much less) or mixed, combining the two forms. For example, LMs of the oral cavity are often microcystic, but those in the armpit or groin are macrocystic or mixed. One hypothesis explaining these differences in size is the density of the connective tissue underlying the cyst during its formation: loose connective tissue would be associated with larger cysts.

LMs can be isolated or syndromic (Table 1). They can compress the neighboring organs or be responsible for seepage of lymph or bleeding when in the form of lymphangiectasia (microcystic superficial mucosal or cutaneous LMs).

**Table 1:** Main syndromes including cystic lymphatic malformations (LMs)

Syndrome (MIM, ORPHA)	Main abnormalities associated with LMs	Gene
Proteus syndrome (MIM 176,920 Orpha 744)	Capillary and venous malformation, asymmetric musculoskeletal hypertrophy, epidermal hamartomas	*AKT1* (S)
CLOVES (MIM 612,918 Orpha 140,944)	Venous and capillary malformation, asymmetric lipomatous and musculoskeletal hypertrophy	*PIK3CA* (S)
Klippel-Trenaunay syndrome (MIM 149,000 Orpha 2346)	Capillary malformation of limb, elongated affected limb, venous malformations (varicose veins) with thrombotic risk	*PIK3CA* (S)
Bannayan-Riley-Ruvalcaba (MIM 158,350, ORPHA 109)	Capillary and venous malformation, arteriovenous malformation, macrocephaly, intestinal hamartomatous polyposis	*PTEN* (G)
CLAPO (MIM 613,089, ORPHA 168,984)	Capillary malformation of the lower lip, asymmetry of face and limbs, partial or generalize overgrowth of one or more body segments	*PIK3CA* (S)
Gorham–Stout syndrome (MIM 123,880, ORPHA 73)	Massive osteolysis associated with proliferation and dilation of lymphatic vessels	
Generalized lymphatic abnormality	Diffuse involvement (pulmonary, hepatic, splenic, etc.)	

S: somatic; G: germline

### Epidemiology

Superficial LMs are more frequent than visceral ones but we lack epidemiological data. The incidence of cervico-facial LMs is estimated at 1.2‰–2.8‰ [5].

### Etiology

The origin of LMs is still not fully elucidated. The embryonic development of lymphatic vessels involves the differentiation of venous endothelial cells into lymphatic endothelial cells under the influence of transcription factors (PROX1, SOX18 and COUPTF2). These cells then migrate and multiply in the surrounding mesenchyme to form the primary lymphatic sacs, which, by expanding, form the primary arborescent lymphatic vasculature under the action of the VEGF-C/VEGFR-3 signaling pathway and angiopoietin-1. Other genes involved in the formation of lymphatic channels and valves include *CCBE1*, *ADAMTS3*, *FOXC2*, *NRP2*, and *GATA2*. Dysregulation could lead to increased lymphangiogenesis and LM formation. Indeed, somatic-activating mutations of *PIK3CA*, coding for the catalytic subunit of protein kinase PI3K, have been identified in some LMs [6]. This excessive activation could disrupt and activate the PI3K/AKT/mTOR cell signaling pathway, which is involved in angiogenesis and lymphangiogenesis but also cell growth and metabolism [7]. Mutations in these signaling pathways are probably due to disturbances leading to the development of LMs, but the precise mechanism is not yet clearly elucidated [6, 8]. Somatic *PIK3CA* mutations have also been identified in various *PIK3CA*-related overgrowth spectrum (PROS) hypertrophic syndromes, sometimes including LMs.

## Classification

The International Society for the Study of Vascular Anomalies separated vascular anomalies into tumors and malformations [9]. LMs belong to vascular malformations (along with capillary, venous and arterial malformations) with slow flow. LMs can be isolated, combined (e.g., capillary-lymphatic or capillary-lymphatic-venous malformations) or associated with syndromes (e.g., Klippel-Trenaunay or CLOVES) [3, 5, 10]. LMs are distinguished from primary lymphedema, which is linked to an anatomic or functional defect in lymphatic vessels, that induces an increase in volume of one or more limbs with accentuation of folds and skin fibrosis, whereas LMs consist of malformed lymphatic cysts. The present recommendations focus on micro-, macro- and mixed LMs.

## Diagnosis

### Antenatal

Fetal ultrasonography and MRI are the imaging tools for the detailed prenatal diagnosis and anatomical evaluation of LMs [11, 12]. LMs must be distinguished from increased nuchal translucency, which represents a frequent delay in lymphatic resorption in trisomy 21 and heart disease, etc. but does not indicate the further development of a LM. Most medium to large LMs can be diagnosed from the first trimester of pregnancy, and almost all can be diagnosed on ultrasonography in the second trimester.

The demonstration of LMs justifies management in a Multidisciplinary Center for Prenatal Diagnosis (CPDPN). These structures are the only ones authorized in France to care for fetal disorders since 1994.

The exploration of a fetal LM will generally include a reference ultrasonography, an MRI (after 28 weeks of amenorrhea), then a multidisciplinary discussion with the pre- and perinatal stakeholders (obstetricians, geneticists, radiologists) and the postnatal stakeholders (pediatricians, ENT physicians, etc.). In the absence of a particular association between the LM and chromosomal abnormalities, amniocentesis for karyotype or comparative genomic hybridization is not systematic and is left to the reasoned assessment of the CPDPN physicians [9].

Prenatal imaging aims to characterize the lesion (location, macrocystic, microcystic or mixed, differential diagnoses). Despite the very good resolution of fetal MRI, especially T2-weighted sequences, accurately assessing the presence and/or extension of the microcystic infiltrating component, particularly in the ENT sphere or on the limbs is difficult, which leads to remaining cautious in the anatomical evaluation of the microcystic components. The main differential diagnosis is teratoma, but segmental tissue hypertrophy and abnormalities of the extremities (macrodactyly, abnormally wide space between the toes, or “sandal gap”) must also be sought, which may lead to evoking LM in the context of a PROS syndrome.

### Clinical assessment

**Fig. 2:**
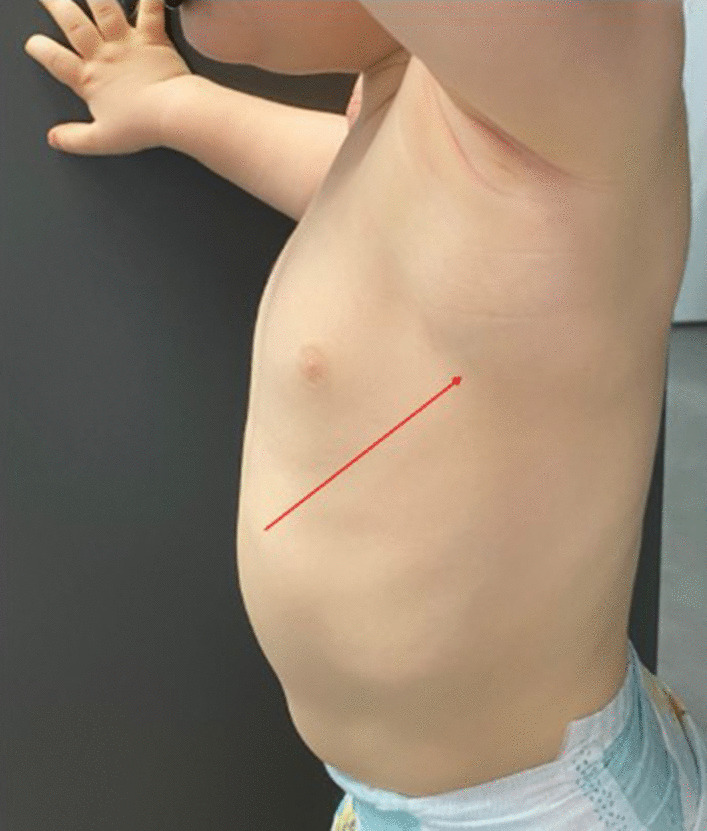
Macrocystic LM of the left armpit that presents as a subcutaneous mass with normal skin color, in a 3-year-old boy

**Fig. 3:**
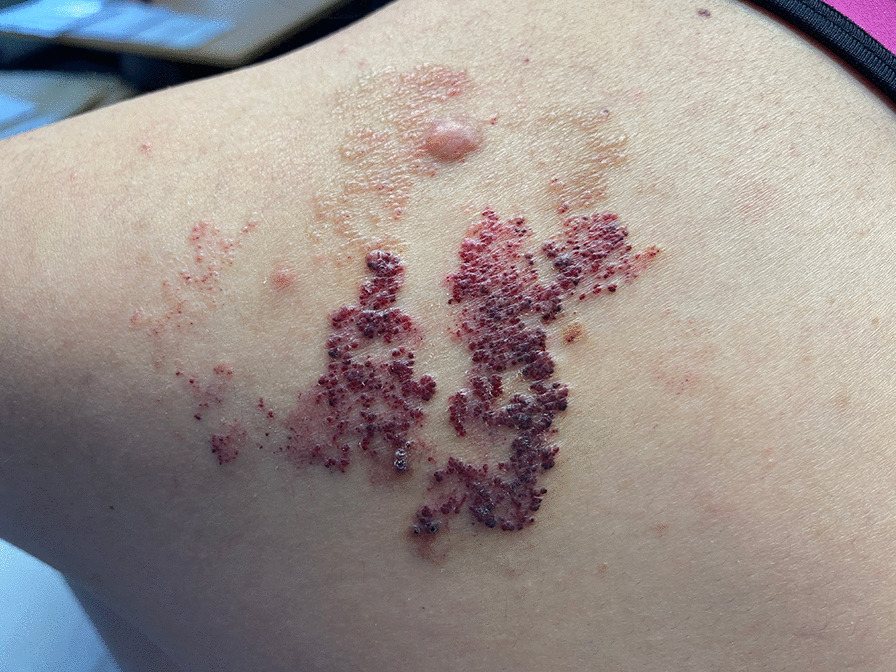
Microcystic LMs of the scapula area in a 13-year-old girl, constituted of cutaneous translucent or hemorrhagic vesicles millimeters in size, scattered or grouped together in plaques, called lymphangiectasias

Superficial or bulky LMs are most often visible at birth. However, they can become apparent later, often during the first 2 years of life, following a viral infections or intracystic hemorrhage [13]. Macrocystic LMs present as subcutaneous, round or lobulated, painless masses, usually several centimeters in diameter, with normal skin color and soft and depressible consistency (Fig. 2). Microcystic LMs can present as comparable subcutaneous swellings, a little firmer, and are then differentiated from macrocystic LMs on imaging. These LM microcysts can also be superficial, epidermal and/or mucous and then appear in the form of translucent or hemorrhagic vesicles millimeters in size, scattered or grouped together in plaques, called lymphangiectasias (Fig. 3) [9]. These can be absent at birth and appear gradually, in childhood or even adulthood.

In all cases, the diagnosis of LMs requires clinical examination and characterization by imaging [11]. Depending on their location, nature and volume, LMs can compress neighboring structures and in particular the upper airways in certain cervico-facial forms, therefore being life-threatening.

The evolution of LMs is marked by inflammatory or infectious episodes, or intracystic hemorrhages, which can be life-threatening on compression (in case of cervico-facial location) and be the cause of pain, hemorrhagic oozing, functional impairment (for example, for orbital LMs) and major deformities. The quality of life of individuals and their families can be strongly affected [6, 9, 14].

### Imaging

Interpretation of imaging data should take into account clinical data. Ultrasonography is the imaging modality for the initial assessment of LMs, especially small superficial lesions [5]. In macrocystic LMs, with hemorrhagic changes, ultrasonography reveals anechoic or heterogeneous liquid cystic lesions, most often compartmentalized with fine septa, which can become heterogeneous, iso- or hyperechoic, sometimes with a liquid–liquid level, in case of intracystic bleeding, by rupture of small vessels of the septa. Conversely, a microcystic LM is characterized by an echogenic infiltration, without hypervascularization on color Doppler ultrasonography, with sometimes a few millimeter-sized cysts.

MRI is the tool of choice for evaluating anatomical extension; characterizing lesions (microcystic and macrocystic), in particular in the event of deep extension; and before considering therapeutic management. It must involve T2 sequences with fat saturation in at least two spatial planes. It can be supplemented with a T1 sequence to identify hemorrhagic changes and visualize infiltration within fatty structures [5, 15]. The injected sequences are used only in case of diagnostic doubt. Angio-MRI has no value in LMs.

Macrocystic malformations are characterized by a juxtaposition of hyperintense cystic structures on T2 septa with possible internal fluid levels, characteristic of intracystic hemorrhage. On T1 sequences, macrocystic malformations are hypo-intense or iso-intense to the muscle, but the septa are more difficult to identify. After administration of the contrast product, the septa within the macrocystic malformation can be enhanced, unlike the cystic components, which allows for distinguishing the macrocystic LM from venous malformations.

LMs with a microcystic component appear as a hyper-intense infiltration on T2-weighted sequences and hypo-intense or iso-intense T1 sequences. Hypodermic infiltrations or within deep fatty structures are much better identified on T2 sequences because of the fat saturation. As on ultrasonography, on MRI, microcystic LMs can appear as a solid lesion, most often infiltrative, and careful examination will sometimes identify small cystic components leading to the diagnosis.

In case of periorbital LM, a cerebral MRI is also recommended to detect associated cerebral venous abnormalities.

Arteriography, venography, lymphography and isotopic lymphoscintigraphy have no value in common LMs. CT-scanning may have some interest in LM involving bones, notably by showing the invasion and deformation caused by microcysts.

### Biopsy

Biopsy is rarely necessary to establish the diagnosis of LMs. However, in case of doubt, the examination shows thin-walled vessels and cysts with empty lumen (hematoxylin–eosin-saffron staining). Lymphatic vessels express the lymphatic markers podoplanin, CD34, LYVE-1 and VEGFR-3 [6]. A biopsy (coupled to a blood sample) can also be used for molecular biology analyses, to search for activating mutations of the *PIK3CA* gene, particularly with LM integrating in a syndromic form (CLOVES or Klippel-Trenaunay syndrome) but also in certain isolated LMs. Fine-needle aspiration biopsy is sometimes performed for diagnostic purposes. It would show a citrine liquid rich in lymphocytes.

### Other explorations

There is no specific biological test for isolated LM. In syndromic or combined forms, consumption coagulopathy must be sought (increased D-dimer level, decreased fibrinogen level). In case of chronic bleeding, one should look for iron deficiency anemia. Finally, a specific assessment can be requested pre-therapeutically.

### Differential diagnoses

The differential diagnoses are mainly congenital cysts (thyroglossal duct cysts, thymic cysts, branchial cysts, bronchogenic cysts and digestive duplications); superficial venous malformations; deep infantile hemangiomas; and other malformations or tumors (mainly teratomas). The microcystic forms can be confused with angiokeratomas, botryomycomas, molluscum contagiosum or condyloma when they are on the genital or perianal areas.

## Natural history

The evolution of LMs is marked by asymptomatic periods, sometimes several years, potentially interspersed with episodes of painful inflammatory flare-ups, superinfections or intracystic hemorrhages of variable frequency. Not all patients have complications or flare-ups and some have a stable form of LM for very long periods.

When occurring, these outbreaks are often painful, resulting in large and sudden increases in volume. They last from 48 h to 1 month, about 10 days on average. They can appear after trauma or with infectious episodes, in particular viral ones.

During these attacks, compression of neighboring organs is possible, which can be life-threatening or induce functional complications. Effusions or hemorrhage are possible (visceral LMs), and intra-abdominal mesenteric locations can also cause intestinal torsion around the cyst. Sex and LM location do not seem to affect the progression and complications. Puberty might be marked by a peak in progression, although the role of hormones has not been demonstrated.

### Specificities of macrocysts

Distinguishing inflammatory outbreaks and infection of macrocysts is difficult because in both cases, the lesions increase in volume, become painful, and can be associated with signs of cutaneous inflammation and fever. Fibrosis secondary to an inflammatory reorganization can explain certain spontaneous regressions (“natural sclerosis”). This spontaneous regression would be more frequent for cervical localizations of LMs and would occur in just over 10% of cases and 2 to 24 months after the episode [16, 17]. Spontaneous regression is possible in up to 16% of cases [18].

Intracystic hemorrhage is also possible: the swelling then becomes suddenly tense, painful, and potentially bluish but without fever or inflammatory signs.

### Specificities of microcysts

Microcystic LMs may present as swellings under normal skin or as translucent or hemorrhagic vesicles millimeters in size, scattered or grouped in plaques, called lymphangiectasia. They may be covered with verrucous hyperkeratosis and may be complicated by lymph oozing and chronic bleeding.

The spontaneous evolution generally is toward progressive aggravation, with an increase in the number of vesicles, thickening, increased oozing and bleeding. Oral locations may be malodorous due to bacterial colonization.

Orbital microcystic LMs pose specific problems (exophthalmia, diplopia, pain) and require the advice of an ophthalmologist specialized in orbito-palpebral pathology. The main complication is compressive optic neuropathy, which can occur acutely (bleeding or more rarely infection) or chronically: decreased visual acuity, relative afferent pupillary deficit, papilledema, visual field alterations and dyschromatopsia.

## Therapeutic possibilities

Some LMs are barely symptomatic or not at all, in which case no therapy, with a follow-up proposal, is the most appropriate management. If treatment is necessary, the different options can be successive or concomitant, and decisions must be made in multidisciplinary consultation.

Apart from very small LMs that can be completely resected, the goal of treatment is generally not curative but to maintain functionality, control associated symptoms, preserve aesthetic integrity and quality of life, and prevent aggravation.

### Interventional radiology (sclerotherapy)

Sclerotherapy is the standard first-line treatment for most LMs, generally performed under general anesthesia, although it is possible under local anesthesia for certain products [19]. It is significantly more effective in macrocystic LMs [20] than microcystic LMs [21]. Sclerosants, generally used as monotherapy, are tissue irritants that cause endothelial damage and inflammation, thus leading to fibrosis and vascular obliteration [22, 23]. They are injected after aspiration of the cystic content and opacification, for a volume of 30% to 50% of the aspirated cystic content. The secondary inflammatory reaction can be painful and/or dangerous (eye, airways). Efficacy is estimated after at least 4–6 weeks, and several sessions are sometimes necessary [23].

Several sclerosants can be used: sodium tetradecyl sulphate, sodium morrhuate, OK-432, polidocanol or lauromacrogol 400 (3% Aetoxisclerol®), absolute ethanol, doxycycline and bleomycin. Each sclerosant has its advantages and disadvantages, and their use is also linked to the center’s experience. For example, absolute ethanol is potent but generates a risk of cardiac arrest if it enters systemic circulation [22]. Bleomycin can have severe side effects and its dosage should be carefully calculated, but it is particularly useful in orbital LMs because of the weak inflammatory reaction it generates.

The side effects of the most commonly used sclerosing agents are similar and include swelling, pain, skin ulcerations and nerve paralysis. The risk of a serious complication (nerve damage, skin necrosis, pulmonary vasospasm, arrhythmia or cardiopulmonary collapse) is in the order of 0 to 3% [22].

Sclerotherapy is the first-line treatment for macrocytic LMs, and the sessions can be repeated several times for a complete result, generally spaced out by at least 3–6 months.

### Surgery

In most cases, complete surgical removal of LMs is difficult or even impossible because of infiltrating lesions that are difficult to identify [24]. Therefore, surgery is generally partial and suspensive and usually second-line treatment [25]. It can be considered for well-limited macrocystic cervical LMs or microcystic LMs to reduce the volume of deforming and troublesome lesions [26, 27]

### Physical treatment

Physical treatment (electrocoagulation, laser treatment, radiofrequency therapy, etc.) is useful for superficially destroying lesions, stopping bleeding, and reducing redness. It must be repeated and is only suspensive [27, 28]. Lingual microcystic LMs is a classic indication. For cutaneous lymphangiectasia, CO_2_ laser treatment is safe and transitory effective but painful. Adverse effects are usually minor and infrequent and include dyspigmentation and mild scarring [29]. Topical sirolimus is under clinical evaluation for these superficial cutaneous and mucosal LMs (NCT03972592, NCT04128722).

### Physiotherapy

Physiotherapy includes compression and lymphatic drainage. Compression has not been found effective in LMs [30]. Several articles mention the use of compression garments/bandages but do not quantify the advantages (pain, quality of life) or disadvantages (e.g., acceptability). Manual lymphatic drainage has not been found effective in LMs, but its very good tolerance and the well-being it can provide can sometimes improve quality of life.

### Medical treatment

Sirolimus (or rapamycin) inhibits lymphangiogenesis by interfering with the PI3K/AKT/mTOR signaling pathway. It has been used in complicated LMs for 10 years [31–39]. Many studies have confirmed its effectiveness, albeit partial (up to 90%), delayed (after 2 weeks to 6 months of treatment) and suspensive. It is effective for symptoms (pain, bleeding, lymphorrhea) and reducing the volume of the LM, although never completely [34]. The duration of treatment is often long, and there are no decision criteria for its discontinuation apart from adverse effects. Regular clinical and biological monitoring (approximately 1 month after treatment initiation, then every 2–3 months) is required. Sirolimus trough concentration should be measured for dose adjustment (target concentration approximately 3–12 ng/ml) Drug interactions should be routinely checked [34–39].

Sildenafil is a potent and selective inhibitor of phosphodiesterase type 5. A few small series have suggested some efficacy but without further confirmation [23, 40]. Therefore, sildenafil is not indicated, except in very specific last-resort situations.

Propranolol is a non-selective beta-blocker that induces peripheral vasoconstriction and endothelial cell apoptosis and is effective for infantile hemangioma. It has not been found effective in LMs [41, 42].

Bevacizumab is a monoclonal antibody active against VEGF-A, with an effect on lymphangiogenesis. Bevacizumab has been the subject of few clinical cases, systemically and as a sclerosing agent [23]. Hence, there is not enough evidence to recommend it in LMs.

Alpelisib is a targeted therapy that directly inhibits the PIK3. However, somatic activating mutations of the *PIK3CA* gene have been identified in LMs integrated in syndromes with hypertrophy of the underlying soft tissues (CLOVES and Klippel-Trenaunay) as well as in certain isolated LMs. A pilot study reported promising results, and this molecule is undergoing clinical evaluation (efficacy and safety profile) for syndromic forms [43].

## Therapeutic management

LMs must be treated by physicians/surgeons integrated in multidisciplinary teams (medico-surgical and psychosocial). Several LM cases can be distinguished.

### Neonatal forms

These very large, macrocystic or mixed LMs are often detected in utero. They can generate dystocia and respiratory distress in the newborn in the event of cervico-facial involvement. An EXIT procedure is indicated in case of foreseeable obstruction during childbirth [44, 45]. However, a tracheotomy is still sometimes necessary, and this possibility should be mentioned during the prenatal consultations. Once the airways are secure, sclerotherapy is indicated as first-line therapy after a control MRI, usually performed within the first 3 months of life. In the case of giant LMs, combining sirolimus (for 12–24 months) with one or more sclerotherapy sessions has given very satisfactory results in several cases [46]. Recently, sirolimus was given as antenatal treatment for a voluminous LM diagnosed in utero, providing promising results that need to be confirmed [47].

### Macrocystic LMs

Sclerotherapy is the first-line treatment in case of discomfort. It must be programmed, outside the context of the emergency. Surgery is a second-line treatment and requires prolonged hospitalization for post-operative care. Therapeutic abstention is possible in the case of small and non-troublesome macrocystic LMs, which can regress spontaneously because of a spontaneous inflammatory phenomenon.

### Microcystic and mixed LMs

These are more difficult to treat because of the infiltration of tissues by innumerable small cysts and require a therapeutic strategy including a combination of repeated treatments that rarely lead to sufficient regression to be satisfactory. Therapeutic abstention is possible for small lesions that are not at all troublesome.

Sirolimus is the disease-modifying treatment of choice. It should be started early in life (early childhood) to prevent the increase in volume of the LM. If started after age 6 years, it has shown efficacy for pain, bleeding, lymphorrhea and partial efficacy for malformation volume [35, 39, 46–50]. Sclerotherapy is limited; bleomycin has been used with some positive results [51]; however, the mode of administration and cumulative toxicity limit the therapeutic possibilities.

Surgery is possible but rarely complete, with high risk of recurrence of LM on underlying scars. However, a partial reduction might be useful to reduce a deforming LM, even though delayed healing with oozing is often observed.

Superficial microcystic components can be treated with CO_2_ laser or radiofrequency for the mucous membranes, with CO_2_ lasers or pulsed dye laser, or with topical sirolimus = or > 0.1% for cutaneous lymphangiectasis.

The treatment of orbital LMs depends above all on their visual impact. In the event of acute proptosis (intracystic hemorrhage), surgery may be necessary with major visual impact (reduction of the lesion volume by puncture, or partial/total resection as a last resort). In some cases, bony orbital decompression may be necessary. In the absence of acute complications, the decision is according to the ocular impact, pain, induced aesthetic discomfort and, more generally, the impact of the LM on quality of life. These LMs may be treated with sclerotherapy, sirolimus, or their combination. Surgery, which is complex, is rarely complete [52]

### Syndromic LMs

When LMs are part of a complex syndrome (Proteus, PROS including CLOVES and Klippel-Trenaunay), the management is complex and combines surgery, sclerotherapy, anticoagulants for venous abnormalities with associated thrombosis, physiotherapy etc. Sirolimus and targeted anti-PI3K therapies are increasingly used [53–58].

### Inflammatory flare-ups

No specific study has compared the different therapies. Analgesic treatment is always indicated (paracetamol then tramadol/paracetamol + codeine depending on the age). In case of fever and suspected infection, probabilistic antibiotic therapy with a fairly broad spectrum is proposed (amoxicillin + clavulanic acid or josamycin for 6–10 days). A high-dose corticosteroid (prednisone or prednisolone, 0.5–2 mg/kg/day) is usually prescribed in case of inflammation for 3–6 days. Antibiotic prophylaxis has sometimes been proposed [59]. Drainage of a macrocyst combined with sclerotherapy is also possible.

### Alternative and complementary medicines

In addition to conventional medicine, “alternative and complementary” medicines are possible and often useful for LMs. They should in no way replace conventional medicine or the follow-up of patients by multidisciplinary medical teams.

With lack of any study validating the superiority of such alternative medicines compared to placebo, they seem to constitute an added value in improving well-being, alleviating pain, acting on inflammatory flares, regulating emotions and sleep disorders, preventing depression and stress, or helping with school integration, accepting one's differences and better living with stares from the public. They allow for better mobilizing the psychosomatic resources of patients and take part in a global approach. Such alternative therapies include body and mind therapies (medical hypnosis, meditation, sophrology, the Surrender method, etc.), manual therapies (osteopathy, chiropractic, massages, drainage, etc.), comprehensive approaches based on theoretical bases that are specific to them (acupuncture, homeopathy, etc.) and biological therapies, which are based on natural products (phytotherapy, etc.). These products, if taken orally, can interact with medications, which needs to be rigorously checked.

### Psychological support

The complex forms of LMs can be difficult to live with for the patient and the family and justify at least a proposal for a psychological interview with, if necessary, prolonged follow-up (management of self-esteem, anxiety and pain, stares from the public, etc.).

### Social support

Depending on local or national legislation, various aids may be possible (adaptation of the workspace, school aids, suspension of the professional activity of a parent to take care of the child, etc.). The patient or family must be referred to the social service department of the hospital or patient associations.

### Monitoring

Patients must be informed of the chronic nature of the pathology, the need for prolonged follow-up, and the generally incompletely curable nature of the lesions [22]. The frequency of follow-up depends on the intensity of the damage; in all cases, the involvement of the attending physician and a specialized multidisciplinary team is essential, especially during adolescence. The French rare diseases network are organized in such a way as to systematically propose a transition between pediatric and adult care. This transition generally takes place around 18 years of age, and the patients are directed towards adapted adult care.

### Pregnancy

Pregnancy does not seem to significantly affect the evolution of LMs. However, cases of inflammatory attacks and an increase in volume of the LM have been mentioned after delivery. Basic treatments should be avoided as much as possible during pregnancy. Sirolimus, for example, should be suspended a few weeks before planning conception and not be administrated during pregnancy and breastfeeding.

### Genetic counseling

In isolated LMs and syndromic forms, somatic (mosaic) mutations have sometimes been identified, affecting the *PIK3CA* gene. This situation considerably reduces the risk of recurrence in the offspring of an affected individual or any siblings, and thus parents can be reassured.

## Conclusion

This work summarizes knowledge, the state of the art and the main indications for assessment and management of LM in 2022. It confirms the decreasing importance of surgery in favor of sclerotherapy and other drug therapies. More than ever, decisions regarding LM patients must be made during multidisciplinary meetings and by teams accustomed to their care.

## Data Availability

Data sharing not applicable to this article as no datasets were generated or analyzed during the current study.

## Abbreviations

*ADAMTS3*: ADAM metallopeptidase with thrombospondin type 1 motif 3
LM: Cystic lymphatic malformation
PROX1: Prospero homeobox 1
SOX18: SRY-Box transcription factor 18
COUPTF2: COUP-TFII (COUP transcription factor 2)
PIK3CA: Phosphatidylinositol-4, 5-bisphosphate 3-kinase catalytic subunit α
PI3K: Phosphoinositide 3-kinase
VEGF: Vascular endothelial growth factor
VEGFR: Vascular endothelial growth factor receptor
*CCBE1*: Collagen and calcium binding EGF domains 1
*FOXC2*: Forkhead box C2
*NRP2*: Neuropilin 2
*GATA2*: GATA binding protein 2
CLOVES: Congenital lipomatous overgrowth, vascular malformations, and epidermal nevi
PROS: PIK3CA-related overgrowth spectrum
MRI: Magnetic resonance imaging
CPDPN: Centre pluridisciplinaire de diagnostic prénatal (multidisciplinary center for prenatal diagnosis)
LYVE-1: Lymphatic vessel endothelial hyaluronan receptor 1
EXIT: Ex-utero intrapartum therapy
